# Ophthalmology

**Published:** 1894-09

**Authors:** Alvin A. Hubbell


### OPHTHALMOLOGY.

BY ALVIN A. HUBBELL, M. D.

### THE ABUSE OF COLLYRIA.

DE WECKER, (*La Médecine Moderne*, May 9, 1894; *Therapeutic Gazette*, July, 1894,) after referring to the necessity of having sterile collyria, is of the opinion that it is unnecessary after every case of extraction to instil atropine on the second or third day and to continue these instillations until recovery is complete, believing that the most perfect cures are observed when, after an extraction properly performed, there is no instillation of collyria.

The use of impure collyria in the treatment of ulcers of the cornea is productive of serious results, and he thinks that ulcers should be treated (1) by careful disinfection of the eyelids and particularly of the palpebral edges; (2) by curetting the ulcers and spraying them, so as to remove all infected parts; (3) by injections of several drops of a solution of sublimate (1 to 2,000) under the conjunctiva in the region of the diseased part; and (4) by the rigorous application of a compressing bandage without the use of collyria.

In the discussion which followed, Armaignac agreed with De Wecker on the abuse of collyria, and especially on the manner of using them when given to poor patients who had no means of keeping them clean. Dufour believes that De Wecker goes too far in attempting to banish collyria, especially atropine. Kalt believed that pilocarpine and eserine should not be used in ulcers of the cornea, but, where there is iritis, atropine should be used. He thinks that the septic condition of the collyria is unimportant, for these are not, in general, more septic than the liquids of the cul-de-sac. The conclusions of Kalt in regard to the uselessness of attempting to obtain aseptic collyria were vigorously disputed by

Darier and Chibret. In the discussion the abuse of cocaine and its deleterious effect on the cornea received prominent attention.

———

TRIKRESOL AS AN ANTISEPTIC FOR COLLYRIA.

E. A. de Schweinitz, Ph. D., Washington, D. C., (*Therapeutic Gazette*, July, 1894,) says the frequent contamination of collyria with harmful bacteria has been pointed out by Francke and others, and again emphasized by recent papers which he has prepared in conjunction with Dr. G. de Schweinitz. Up to the present time, the chemical sterilization of such solutions has been rather unsatisfactory, owing to the strength of the carbolic acid, corrosive sublimate or mercury cyanide, which must be used to insure disinfection ; hence, it occurred to him that trikresol might be useful.

Trikresol is a mixture of the ortho-, meta- and para-methyl phenols, or ortho-, meta- and para-kresols, and contains the hydroxyl group, which seems to be a characteristic of antiseptics and germicides. Its irritating action is restrained, although its disinfecting properties are not interfered with, by the presence of the methyl group in the molecule.

A 1 to 1,000 solution might be used for the preparation of the collyria. The three drugs most liable to contamination in a short time are cocaine, atropine and eserine, and, of these, solutions were made in the following strengths : hydrochlorate of cocaine, four per cent. ; eserine, one grain to the ounce ; atropine, four grains to the ounce. Trikresol water was used as the solvent. These solutions were placed in ounce glass-stoppered bottles, no particular care being used to protect them from dust, and they were allowed to stand for a week in a loosely closed closet in the laboratory. At the end of this time the bottles were opened without flaming their mouths, and cultures made from the solutions, but no growth appeared upon the media. The bottles were then allowed to stand uncorked for a week in a closet which stood just in front of the window. This closet was opened four or five times a day, and no care was taken to prevent contamination with dust. At the end of this time the collyria were still clear, and cultures from them showed no growth. The next day happened to be a breezy one, and, as the door of the closet was open, the dust from the street had free access to the open bottles. Though the solutions themselves still appeared unaffected, cultures from the eserine and cocaine yielded a slight growth, but the atropine remained sterile. This test was much more severe than any to which the collyria, preserved even with the slightest care, would be submitted.

He would suggest, therefore, that it would be advantageous in making up collyria to use trikresol water (1 to 1,000). In addition to the usual solutions kept in the ordinary treatment case, there should also be a small vial of trikresol water for rinsing the pipettes after use. By this method, he thinks that the fungous and bacterial growths often found in collyria might be prevented, as well as any eye-complications resulting from the use of a contaminated solution. These collyria have now been prepared for three months, and no growth, fungous or otherwise, is apparent in any of them, and no growth is yielded by cultures.

---

### BLINDNESS FROM OPHTHALMIA NEONATORUM.

DR. BOERNE BETTMAN, (*Journal of the American Medical Association*, May 19, 1894; *Therapeutic Gazette*, July, 1894,) bringing this matter before the Chicago Medical Society, contributes the following information:

If we consult the United States census from the year 1850 to date, we learn that the number of blind enumerated during the previous decades is as follows: 1850, 9,794; 1860, 12,658; 1870, 20,320; 1880, 48,928; 1890, 50,411.

If compared with the population, which increased from 23,191,876 in 1850 to 62,622,250 in 1890, we obtain the following ratios:

Number of blind to 1,000,000 of population: 1850, 422; 1860, 403; 1870, 527; 1880, 976; 1890, 805.

As we are especially concerned about the State of Illinois, it will be interesting to ascertain the number of blind allotted to us in the records quoted. The following table comprises both the total number of blind for each period and their ratio to 1,000,000 of the population.

Number of blind in the State of Illinois: 1850, 264; 1860, 476; 1870, 1,042; 1880, 2,615; 1890, 2,834.

Number of blind to 1,000,000 inhabitants: 1850, 310; 1860, 278; 1870, 410; 1880, 850; 1890, 741.

It will be observed that the proportion of these sadly afflicted has greatly increased, notably so in 1880, and again decreased in 1890. These apparent inaccuracies are due to the methods of enumeration adopted, and are fully explained in the following words of Dr. Wines, quoted from the Report of the Board of Public Charities of Illinois, 1892:

> With regard to the so-called "defective" classes, it should be known that Dr. Wines, in 1880, supplemented the enumerators' returns by correspondence with physicians, who added many names to the lists. This correspondence was not renewed in 1890, which accounts, at least in a large degree, for the seeming slight falling off in the ratio in 1890.

It is also established that proper prophylaxis, notably Credé's method, in hospital practice and in all cases where known infection exists, and milder prophylaxis in private practice generally, is capable of reducing the number of cases of this disease to a minimum.

Finally, it is established, on the one hand, that failure promptly to recognize and properly to treat this inflammation results in disastrous consequences; while, on the other hand, if suitable measures are quickly and thoroughly instituted, before corneal complications arise, the majority of cases will be brought to a successful termination. The exceptions to this rule are those cases which from the beginning assume a diphtheritic type, or as, for example, Dr. Randall has shown, those which, probably from some inherent malignancy not yet well understood, go on to destruction in spite of the best care and the most scientific treatment.

---

PURULENT OPHTHALMIA: TREATMENT, ETIOLOGY AND PROPHYLAXIS.

Abadie (*La Médecine Moderne*, May 12, 1894; *Therapeutic Gazette*, July, 1894,) points out that, although in laboratory experiments weak solutions of sublimate, carbolic acid, iodoform, naphthol, resorcin and permanganate of potassium destroy the virulence of the gonococci, they do not accomplish this result when this microbe is found in the living tissue of the conjunctiva. All substances are then powerless to avert the evil. They have not a really curative action; they can only arrest to a limited extent the fatally progressive evolution of purulent ophthalmia. Nitrate of silver, on the contrary, has a truly specific and consequently truly curative effect on the gonococci, even when they develop in their favorite element, the human conjunctiva. Sometimes, if it is used too soon, and in the earlier stages of the disease, it causes a very unfortunate change in the character of the inflammation, which assumes a diphtheritic aspect. As a general thing, the strength of the solution should not exceed three per cent., but the application should be thorough, so that the entire surface of the conjunctiva is reached, and the superficial slough which results should be entirely removed before a second application is made. He thinks that boracic acid, sublimate and permanganate of potassium (1 to 1,000) are about equal in their effects, but prefers, during the intervals of the active applications of nitrate of silver, a solution of the same drug (1 to 1,000) in continuous sprayings, as recently recommended by Burchardt.

---

### THE THERAPEUTIC VALUE OF WEAK LENSES.

DR. F. C. HOTZ, of Chicago. (*Journal of American Medical Association*, Vol. XXII., No. 13, and *Ophthalmic Review*, June, 1894.) Admitting that while formerly patients with weak eyes have often received unnecessary medicine, they now often receive superfluous spectacles, Hotz yet cannot agree that the line of usefulness should be drawn against glasses of very low degree.

The mere fact that in a large number of cases of eye discomfort low degrees of refractive error are found, proves nothing, since we know that slight departures from emmetropia are so very common in the human eye. And even the mere statement that the patient was relieved, is in itself not sufficient evidence of the curative power of these glasses.

But if all local or distant conditions, which we know to produce ocular distress independent of refractive errors, are absent, or have been removed without restoring comfort to the eye, if the glasses are used under precisely the same conditions after the glasses have been prescribed as they were used before; or if an irritability of the eyes has resisted all treatment, but is speedily relieved by glasses, then, and then only, we are warranted in attributing the relief of the eyes to the correction of the existing slight refractive errors.

Such cases are exceptional, but he has collected from his case-book the notes of fifty, in which sphericals of less than 0.75 degree, or cylinders of 0.25 degree or sphero-cylindrical combinations of low strength were prescribed. Of the fifty cases treated with weak lenses, thirty patients have reported good results, one patient has reported a negative result and nineteen have not been heard from. Even if all those not heard from are regarded as failures, the results, successful in sixty per cent. of the cases, are gratifying; and proof is afforded that the therapeutic value of these weak lenses is not mythical, but real.

It must not be forgotten that the eye is most successful in correcting, by accommodative efforts, the optical disturbances produced by slight degrees of hypermetropia or astigmatism, while in the higher degrees of these refractive errors it does not succeed, and therefore abandons the effort. The moderate degrees, therefore, are the prevailing errors of refraction found in patients complaining of the symptoms of eye strain.

The degree of ametropia does not constitute the principal condition in the production of distressing symptoms, and experience does not sustain the statement that hypermetropia or astigmatism less than 0.75 degree seldom causes discomfort or headache. The state of health, the condition of the nervous system, the occupation, are very important factors, and frequently account for the fact that in many persons a slight hypermetropia or astigmatism causes great distress, while other persons never experience the slightest discomfort from ametropia the same in kind and degree.

Many eyes can endure a great amount of strain with impunity, while other eyes are so constituted that their powers of endurance are quickly exhausted. One person may need glasses for the correction of a small amount of ametropia, while in another the correction of a much higher degree is unnecessary, and glasses would be superfluous. We cannot draw the line at a certain amount of ametropia, but should correct it, no matter how slight in degree, whenever it leads to disturbances for which eye strain constitutes the most frequent cause.

---

### IMMEDIATE CAPSULOTOMY FOLLOWING THE REMOVAL OF CATARACT.

L. Webster Fox, M. D., of Philadelphia, (*Medical Bulletin*, July, 1894,) says that after the delivery of the lens (cataract) and all cortical matter is washed out of the anterior chamber, he proceeds with the rupturing of the posterior capsule. The instrument used is a gold-enameled hook, made as delicate as is consistent with keeping its shape. It is of malleable steel, so that it may be bent to any angle, which he finds is convenient, especially when the eye of the patient lies deep in the orbit. The hook is passed into the anterior chamber and behind the lower pupillary margin of the iris on its flat side. It is then rotated backward, hooked into the capsule, drawn gently upward to the mouth of the incision, rotated on its flat side again, and then taken out of the chamber. By this means the capsule is torn and the vitreus presses forward between the rent. Very little or no vitreus shows at the mouth of the wound. If it does, he snips it off.

When the operation is performed after the simple method (without iridectomy), the same manipulation is carried on with but one exception, and that is, the line of incision is not so long The ophthalmostat is removed and the eyeball again irrigated with the hydrostatic eye-douche, followed by dropping one drop of sterilized atropia solution into the eye; the lids closed and thickly anointed with vaselin, which has been sterilized by boiling; over this, specially devised eye-pads, which have been sterilized by heat, held in place by adhesive strips, which keep the bandages securely fixed, permitting the patient to change his position in bed as often as is desirable. In twenty-four hours the dressings are removed and both eyes bathed with warm water and irrigated with the sulpho-carbolate solution, another drop of atropia applied and similar eye-pads, adjusted with as much care as at the primal, operation; and so continued from day to day until the eye is out of danger.

He has been in the habit of performing this operation in alternating cases for ten years. In those patients upon whom the operation was performed, he had to repeat a needle or capsulotomy (scissors) in about 15 per cent. of the cases; where it was not performed, in about 75 per cent. In the 15 per cent. of the cases where it did not succeed, he can only attribute it to a very thick posterior capsule, the vitreus receding after closing of the eyeball, and thereby not keeping the capsule separated, but practically closing again. His experience has led him to believe that there is less danger of inflammation of the eyeball in immediate capsulotomy than in a subsequent operation.

---

### A SUGGESTION OF AN OPERATION TO CORRECT ASTIGMATISM.

Dr. W. H. Bates, of New York, (*Archives of Ophthalmology*, January–April, 1894,) after reporting several cases, makes the following propositions: (1) A corneal incision lengthens the radius of curvature of that corneal meridian which is at right angles to the line of the incision, and does not flatten any other meridian. The astigmatism produced is a regular astigmatism, and is corrected by a convex cylinder at an axis parallel to the line of the incision. (2) The immediate result is greater than the ultimate result. (3) The astigmatism produced is permanent after a length of time—at least a month after the cornea has healed. There may be at first 3 d. of astigmatism produced. At the end of a month, there may be 2 d. At the end of three months, the astigmatism may still be 2 d., and this amount of astigmatism will be permanent. (4) The amount of astigmatism produced is greater the nearer the incision is to the center of the cornea. As much as 9 d. can be produced. (5) Mixed astigmatism occurs: (*a*) temporarily; (*b*) with incarceration of the iris.

A study of Case III. would show that proposition 1 still holds true, and that the myopia is due to other causes than the cornea. The myopia is due to swelling of the lens or to lengthening of the eyeball.

*The Operation Suggested.*—Incisions of the cornea are made at right angles to the most convex meridian. The amount of correction can be regulated by the number, depth and location of the incisions.

The operation promises a permanent effect. The risk to the eye is not great. It is not as dangerous an operation as the operation of iridectomy, which is usually performed without accident. Incarceration of the iris must be avoided to prevent the development of myopia.

---

### CATARACT: MORPHINE, HYPODERMICALLY, AS A MEANS TO PREVENT PROLAPSE OF THE IRIS IN SIMPLEX EXTRACTION.

DR. EUGENE SMITH, of Detroit, (*Archives of Ophthalmology*, January–April, 1894,) to prevent prolapse of the iris, proceeds as follows for simple extraction of cataract: After the toilet of the eye subsequent to the operation, he drops a one-grain solution of eserine (salicylate of physostigmine) in the conjunctival sac, gives a hypodermic injection of one-quarter grain of morphine sulphate, waits a few minutes, then lets the patients go to bed, where it is his choice that they remain from twenty-four hours to two days. Eight hours after the first hypodermic injection of morphia he gives another, and the following morning a third. This generally keeps the pupil contracted, *ad maximum*, from thirty-six to forty-eight or more hours.

The morphine injection also relieves the discomfort immediately following the operation, produces tranquility, checks a cough if present, and does away with involuntary traction of the recti or orbicularis muscles. Given hypodermically, morphine does not produce vomiting. Whether the effect of morphine in producing contraction of the pupil be due to its sedative action upon the sympathetic nervous system, in consequence of which the capillary vessels of the iris become somewhat engorged, and the pupil contracts strongly, or the contraction be due to a stimulation of the motor-oculi centers, he does not know ; but that it seems to fulfil many indications, and have a most happy effect, he has demonstrated to his own satisfaction in the sixteen cases in which he has employed it.

